# HIV exposed seronegative (HESN) compared to HIV infected individuals have higher frequencies of telomeric Killer Immunoglobulin-like Receptor (KIR) B motifs; Contribution of KIR B motif encoded genes to NK cell responsiveness

**DOI:** 10.1371/journal.pone.0185160

**Published:** 2017-09-22

**Authors:** Elise Jackson, Cindy Xinyu Zhang, Zahra Kiani, Irene Lisovsky, Benjamin Tallon, Alexa Del Corpo, Louise Gilbert, Julie Bruneau, Réjean Thomas, Pierre Côté, Benoit Trottier, Roger LeBlanc, Danielle Rouleau, Cécile Tremblay, Christos M. Tsoukas, Jean-Pierre Routy, Xiaoyan Ni, Tsoarello Mabanga, Nicole F. Bernard, on behalf of the Montreal Primary Infection Study Group

**Affiliations:** 1 Research Institute of the McGill University Health Center (RI-MUHC), Montreal, Quebec, Canada; 2 Department of Microbiology and Immunology, McGill University, Montreal, Quebec, Canada; 3 Division of Experimental Medicine, McGill University, Montreal, Quebec, Canada; 4 Centre de Recherche du Centre Hospitalier de l’Université de Montréal (CR-CHUM), Montreal, Quebec, Canada; 5 Department of Family Medicine, Université de Montréal, Montreal, Quebec, Canada; 6 Clinique Médicale l’Actuel, Montréal, Québec, Canada; 7 Centre Hospitalier de l’Université de Montréal (CHUM), Montreal, Quebec, Canada; 8 Clinique Médicale du Quartier Latin, Montreal, Quebec, Canada; 9 Clinique OPUS, Montreal, Quebec, Canada; 10 Département de Microbiologie, Infectiologie et Immunologie, Montreal, Quebec, Canada; 11 Chronic Viral Illness Service, MUHC, Montreal, Quebec, Canada; 12 Division of Clinical Immunology, MUHC, Montreal, Quebec, Canada; 13 Division of Hematology, MUHC, Montreal, Quebec, Canada; Emory University School of Medicine, UNITED STATES

## Abstract

Previously, we showed that Killer Immunoglobulin-like Receptor *(KIR)3DS1* homozygotes (hmz) are more frequent in HIV exposed seronegative (HESN) than in recently HIV infected (HIV+) individuals. *KIR3DS1* encodes an activating Natural Killer (NK) cell receptor (NKR). The link between *KIR* genotype and HIV outcomes likely arises from the function that NK cells acquire through expression of particular NKRs. An initial screen of 97 HESN and 123 HIV+ subjects for the frequency of *KIR* region gene carriage observed between-group differences for several telomeric *KIR* region loci. In a larger set of up to 106 HESN and 439 HIV+ individuals, more HESN than HIV+ subjects were *KIR3DS1* homozygotes, lacked a full length *KIR2DS4* gene and carried the telomeric group B KIR haplotype motif, *TB01*. *TB01* is characterized by the presence of *KIR3DS1*, *KIR2DL5A*, *KIR2DS3/5* and *KIR2DS1*, in linkage disequilibrium with each other. We assessed which of the *TB01* encoded KIR gene products contributed to NK cell responsiveness by stimulating NK cells from 8 HIV seronegative *KIR3DS1* and *TB01* motif homozygotes with 721.221 HLA null cells and evaluating the frequency of KIR3DS1^+/-^KIR2DL5^+/-^, KIR3DS1^+/-^KIR2DS1^+/-^, KIR3DS1^+/-^KIR2DS5^+/-^ NK cells secreting IFN-γ and/or expressing CD107a. A higher frequency of NK cells expressing, versus not, KIR3DS1 responded to 721.221 stimulation. KIR2DL5A^+^, KIR2DS1^+^ and KIR2DS5^+^ NK cells did not contribute to 721.221 responses or modulate those by KIR3DS1^+^ NK cells. Thus, of the *TB01* KIR gene products, only KIR3DS1 conferred responsiveness to HLA-null stimulation, demonstrating its ligation can activate *ex vivo* NK cells

## Introduction

Natural killer (NK) cells are a lymphocyte subset involved in early defenses to virus infected and transformed cells [1]. They contribute to the elimination of these “altered self” cells, in the absence of prior antigen sensitization, by direct cytotoxicity and by secreting cytokines such as IFN-γ and TNF-α and chemokines such as CCL3, CCL4 and CCL5 [2–5]. NK cells also act to bridge innate and adaptive immunity, by contributing to the activation of T and B cells through dendritic cell activation and cytokine production [6].

NK cell activity is regulated by an array of cell surface receptors. The most diverse of these are the structurally related polymorphic Killer Immunoglobulin-like Receptors (KIR) [7]. The *KIR* gene cluster is located on the long arm of chromosome 19 (19q13.4) within the leukocyte receptor complex [8]. *KIR* genes are organized into group A or B haplotypes [9–11]. The group A haplotypes are comprised of four framework genes present in most *KIR* haplotypes (*KIR3DL3* at the centromeric end, *KIR3DL2* at the telomeric end and *KIR2DL4* and the pseudogene *KIR3DP1* in the middle) plus *KIR2DL1*, *KIR2DL3*, *KIR3DL1*, *KIR2DS4* and *KIR2DP1*. The *KIR2DS4* locus encodes several variants having a frameshift mutation that prevents cell surface expression [12, 13]. These are present at a high frequency in certain populations, such that many individuals homozygous for the *KIR* group A haplotype have no activating KIR (aKIR) [12]. The more diverse group B haplotypes include the framework genes with various combinations of *KIR2DL2*, *KIR2DL5A/B*, *KIR2DS1*, *KIR2DS2*, *KIR2DS3*, *KIR2DS5* and *KIR3DS1* [14–16]. Most *KIR* region haplotypes are composed of one of 3 centromeric and one of 3 telomeric KIR motifs that include combinations of KIR genes in linkage disequilibrium (LD) with each other [17]. The centromeric region is delimited by the framework genes *KIR3DL3* and *KIR3DP1* while the telomeric region is delimited by framework genes *KIR2DL4* and *KIR3DL2* [18].

The engagement of inhibitory KIR (iKIR) by surface major histocompatibility complex class I (MHC-1) or HLA antigens on neighboring cells during development is required for NK cell education, a process that confers NK cells with functional competence [19, 20]. In mature educated NK cells, the engagement of iKIR by HLA results in inhibitory signals. Virus-infected and transformed cells with altered cell surface HLA expression can drive NK cell activation by altering MHC-1 expression that reduces or interrupts inhibitory signaling through iKIR and by inducing ligands that engage activating NK cell receptors (aNKR) [21].

Epidemiologic studies have found that some *KIR and KIR/HLA* genotype combinations are associated with protection from HIV infection in HIV exposed seronegative (HESN) individuals. For example, co-expression of the high expression homozygous *KIR3DL1* genotype KIR3DL1*h/*y and HLA-B*57 occurs at a higher frequency in HESN than in HIV-susceptible seropositive subjects as does the *KIR3DS1* homozygous genotype [22, 23].

*KIR2DS4* codes for an aKIR. Alleles at this locus can be broadly grouped into those encoding cell-surface expressed (KIR2DS4*001-like) and truncated, non-cell-surface expressed (KIR2DS4*003-like) variants [12, 13]. KIR2DS4*001 has been associated with HIV transmission in HIV discordant couples in Zambia, independently of its association with higher HIV viral load in index transmitting partners [24]. Carriage of these alleles has also been associated with poor outcomes such as low CD4 counts and/or high viral load in a cohort of HIV-infected American youth and in HIV+ individuals in Lima, Peru [25, 26]. It is notable that the *KIR2DS4* and *KIR3DL1/S1* genes are in LD with each other and with other *KIR* genes in telomeric *KIR* region motifs [17]. The *KIR2DS4* and *KIR2DS1* genes are in negative LD, suggesting they may be alleles at the same locus [27, 28]. Carriage of *KIR2DS1* and absence of a *KIR2DS4* gene is a hallmark of the telomeric *TB01* motif [17].

KIR2DL5 is presumed to be an iKIR based on its long immunoreceptor tyrosine-based inhibitory motif (ITIM) containing cytoplasmic tail [29, 30]. The ligand for KIR2DL5 remains unknown. The gene encoding KIR2DL5 is duplicated in some *KIR* group B haplotypes [31]. *KIR2DL5A* lies in the telomeric region next to *KIR3DL1/S1*, while *KIR2DL5B* is in the centromeric region [11, 32]. *KIR2DL5* genes mark centromeric and telomeric group B haplotypes [14]. While many *KIR2DL5B* gene products are not cell surface expressed, KIR2DL5A receptors are expressed on the surface of CD56^dim^ NK cells [29]. Based on LD with *KIR2DL5A* and *KIR2DL5B*, *KIR2DS3* and *KIR2DS5* can also be present in the telomeric and centromeric group B haplotypes [33, 34]. The *KIR2DS3* and *KIR2DS5* genes are in negative LD with each other and have been proposed to be allele groups at the same locus [27, 28].

Given the LD between genes located in the telomeric group B *KIR* region and the previously reported higher frequency of *KIR3DS1* homozygotes (hmz) among HESN compared to HIV+ subjects, we investigated the differential frequency of other KIR region genes in these two populations. The *TB01* motif of linked *KIR* genes was found more frequently among HESN than HIV+ subjects. We took advantage of the stochastic expression of *KIR* gene products on the NK cells to investigate the contribution of KIR3DS1, KIR2DL5A, KIR2DS1 and KIR2DS5 to NK cell responses to the HLA null cell line 721.221 (221).

## Materials and methods

### Ethics statement

This study was conducted in accordance with the principles expressed in the Declaration of Helsinki and was approved by the Institutional Review Boards of the Comité d’Éthique de la Recherche du Centre Hospitalier de l’Université de Montréal and the Research Ethics Committee of the McGill University Health Centre. All individuals provided written informed consent for the collection of samples and subsequent analyses.

### Study population

The study population for KIR region typing included a total of 545 individuals, of which 106 were HESN and 439 were HIV-infected individuals enrolled in the Montreal Primary Infection (PI) cohort. HESN were recruited from the St. Luc cohort, a prospective cohort of active HIV-negative injection drug users (IDU) at high risk for HIV acquisition [35] (n = 87), and among HIV-negative partners of serodiscordant couples followed in medical clinics in Montreal (n = 19). Information collected at follow-up visits included assessment of the frequency of high-risk behavior for HIV acquisition, blood draws and monitoring of HIV serostatus. All HESN subjects maintained a negative HIV enzyme immunoassay (HIV EIA) test despite at least five reported HIV exposures. Parenteral exposure was defined as sharing needles with known HIV-infected partners and mucosal exposure was defined as unprotected sex with a known HIV-infected partner. None of the HESN subjects were *CCR5Δ32* homozygotes, a genotype known to confer resistance to HIV infection [36, 37]. The Montreal PI cohort enrolls individuals within 6 months of infection and follows them an average of every 3 months for up to 4 yrs. At each visit CD4, CD8 and plasma viral load measurements are done and peripheral blood mononuclear cells (PBMC) and plasma are frozen and stored.

For functional assays, we studied 8 HIV–uninfected *KIR3DS1* hmz, including 7 with at least 1 *Bw4* allele and 1 who was a *Bw6* hmz with no *Bw4* alleles at the *HLA-A* locus. All the *KIR3DS1* hmz were positive for *KIR2DL5A* and *KIR2DS1* genes, 6 carried a *KIR2DS5* gene and none carried a gene encoding an expressed KIR2DL5B variant. All were negative for *KIR2DS4*. Table 1 shows the HLA and KIR information for each of these 8 study participants.

**Table 1:** KIR/HLA information on KIR3DS1 homozygotes providing cells for functional studies.

Donor	HLA-A	HLA-B	HLA-C	Bw4	KIR2DL5A	KIR2DS1	KIR2DS5
1	01:01/02:03	37:01/46:01	01:02/06:02	Yes	Yes	Yes	No
2	02:01/24:02	39:01/51:01	07:02/07:02	Yes	Yes	Yes	Yes
3	02:01/03:01	27:05/47:01	02:02/06:02	Yes	Yes	Yes	Yes
4	02:05/03:01	44:02/44:02	06:02/07:01	Yes	Yes	Yes	Yes
5	24:02/68:01	18:01/18:01	05:01/07:01	Yes	Yes	Yes	Yes
6	24:02/68:01	18:01/57:01	06:02/07:01	Yes	Yes	Yes	Yes
7	02:01/32:01	27:05/51:01	05:01/14:02	Yes	Yes	Yes	Yes
8	03:01/11:01	07:02/40:02	02:02/07:02	No	Yes	Yes	No

### Genotyping

Genomic DNA was extracted from PBMCs or Epstein-Barr virus (EBV)-transformed cells using a QIAamp DNA blood kit (QIAGEN, Inc., Mississauga, Ontario, Canada). *KIR* region typing was performed on 97 HESN and 123 HIV+ subjects using commercially available reagents (KIR Genotyping SSP kit, OneLambda, Canoga Park, CA) according to manufacturer’s instructions. The presence of the following *KIR* genes was detected: *KIR2DL1-2DL5*, *KIR2DS1-2DS5*, *KIR3DL1-3DL3*, *KIR3DS1* and the pseudogenes *KIR2DP1* and *KIR3DP1*. All subjects carried the framework KIR genes, a *KIR3DL1/S1* and a *KIR2DL2/L3 gene*. The subjects who were *KIR* region typed, as well as an additional 9 HESN and 316 HIV+ subjects for a total of 106 HESN and 439 HIV+ subjects, were typed for generic genotypes at the *KIR3DL1/S1* locus using 2 sets of primers specific for *KIR3DL1* and *KIR3DS1* as previously described [23, 38]. A total of 105 HESN and 438 HIV+ subjects were tested for the presence of a *KIR2DS4* gene and, if present, for KIR2DS4*001-like and KIR2DS4*003-like alleles by either *KIR* region typing and/or using 2 sets of primers specific for *KIR2DS4* and conditions described by Kulkarni et al. [38]. The presence of *KIR2DL5*, *KIR2DS3*, *KIR2DS5* and *KIR2DS1* genes was assessed in 105 HESN and in 431, 321, 321 and 435 HIV+ subjects, respectively, by *KIR* region typing and/or using 2 sets of primers specific for these genes [38]. All subjects positive for a *KIR2DL5* gene were typed for the presence of a telomeric *KIR2DL5A*, a centromeric *KIR2DL5B* gene or both using a modification of methods described by Du et al. [33]. *KIR2DL5A* and *KIR2DL5B* genes were distinguished at 3 single nucleotide polymorphisms (SNP) at positions (-97, -84, and +16) [33]. Additionally, when a *KIR2DL5B* gene was present, the SNP present at position -97 was used to deduce whether it encoded an expressed gene product or one that was epigenetically silenced [32, 39]. Testing for the presence of *KIR2DS3* and *KIR2DS5* genes was performed on all individuals who carried *KIR3DS1*, *KIR2DL5A* or *KIR2DS1* genes to ascertain whether one of these was present, as would be expected of carriers of a canonical *TB01* motif. The presence of non-canonical telomeric motifs (i.e. other than *TA01*, *TA02* or *TB01* motifs) were verified by repeat typing.

### Cells

Cryopreserved HLA-null 221 cells were thawed and cultured in RPMI medium supplemented with 10% Fetal Bovine Serum (FBS); 2mM L-glutamine; 50 IU/mL penicillin and 50 μg/mL streptomycin (R10) (all from Wisent, St Jean Baptiste, QC, Canada). PBMCs were isolated by density gradient centrifugation (Lymphocyte Separation Medium, Wisent) and cryopreserved in 10% dimethyl sulfoxide (DMSO; Sigma-Aldrich, St Louis, MO); 90% FBS (Wisent).

### NK cell activation by 221 cells

Cryopreserved PBMCs were thawed and co-cultured in R10 with 221 HLA-null cells at a 10:1 ratio for 6 hr at 37°C in a humidified 5% CO_2_ incubator. Unstimulated PBMCs were cultured alone as a negative control and PBMCs stimulated with 1.25μg/mL phorbol 12-myristate 13-acetate; 0.25μg/mL ionomycin (P/I) were used as a positive control to ensure that the NK cells were viable and functional. CD107a-BV421 (clone H4A3, BioLegend, San Diego, CA) was added at the start of the stimulation; Brefeldin A (5 μg/ml; Sigma-Aldrich) and monensin (6 μg/ml, Golgi Stop; BD Biosciences, Mississauga, ON, Canada) were added 30 min after starting the co-culture. After stimulation, cells were stained using the UV Live/Dead Fixable Dead Cell Stain Kit (Invitrogen, Burlington, ON, Canada) to assess viability. Nonspecific interactions with antibodies in the staining panel were minimized by using TruStain FcX reagent (BioLegend), as per the manufacturer’s instructions. Cells were surface stained with one of 2 fluorochrome-conjugated antibody panels. Panel 1 included antibodies to the following specificities: CD3-BV785 (OKT3), CD56-BV711 (HCD56, both from BioLegend), KIR3DL1/S1-PE (Z27, Beckman Coulter, Mississauga, ON, Canada) and KIR2DL5-PE-Vio 770 (Miltenyi Biotec, Cambridge, MA). Panel 2 included antibodies to the following specificities: CD3-BV785 (OKT3), CD56-BV605 (HCD56) and KIR2DL1/KIR2DS1/KIR2DS3/KIR2DS5-FITC (HP-MA4, all from BioLegend), KIR2DL1-VioBlue (REA284) and KIR2DL1/KIR2DS1-APC-Vio 770 (11PB6) (all from Miltenyi Biotec) and KIR3DL1/S1-PE (Beckman). After surface staining, cells were fixed and permeabilized using Fix and Perm Kit (Invitrogen) reagents and stained for intra-cellular IFN-γ with anti-IFN-γ-BV510 (B27, BD Biosciences, San Jose, CA). Samples were washed, fixed with 2% paraformaldehyde (Santa Cruz Biotechnology, Santa Cruz, CA), and acquired within 24 hrs.

### Flow cytometry analysis

Between 4.0 x 10^5^ and 1.0 x 10^6^ total events were acquired for each sample using a calibrated LSRFortessa^™^ X-20 flow cytometer (BD). Single stained control beads (CompBead; BD) were used in every experiment to calculate compensation. Boolean gating was used to identify the frequency of KIR3DS1^+/-^ KIR2DL5^+/-^, KIR3DS1^+/-^ KIR2DS1^+/-^ and KIR3DS1^+/-^ KIR2DS5^+/-^ expressing CD3-CD56^dim^ NK cells positive for all possible functional subsets defined by CD107a expression and IFN-γ secretion. For stimulations with 221 cells, PBMCs cultured in R10 served as background controls. All data obtained were corrected for background. Flow cytometry results were analyzed using FlowJo software (V9.8; TreeStar, Ashland, OR).

### Statistical analysis

Statistical analysis and graphical presentation of genotyping results were performed using GraphPad InStat 3.10 and GraphPad Prism 6 (GraphPad Software Inc, La Jolla, CA). Fisher’s exact tests were used to compare proportional between-group differences for selected genes and genotypes in HESN and HIV+ subjects. Results are reported in the following format: (Odds Ratio [95% confidence intervals], p-value) unless otherwise specified. A p-value of less than 0.05 was considered significant. The Holm- Bonferonni method was used to adjust p-values for multiple comparisons. For functional studies, Friedman tests were used to test the significance of differences between the frequency of four within-subject KIR3DS1^+/-^ KIR2DL5^+/-^, KIR3DS1^+/-^ KIR2DS1^+/-^ and KIR3DS1^+/-^ KIR2DS5^+/-^ CD3^-^CD56^dim^ NK cell populations responding to 221 cells. Wilcoxon tests were used to assess the significance of comparisons for within-subject paired data sets for functional NK cell populations.

## Results

### KIR region typing

**Fig 1:**
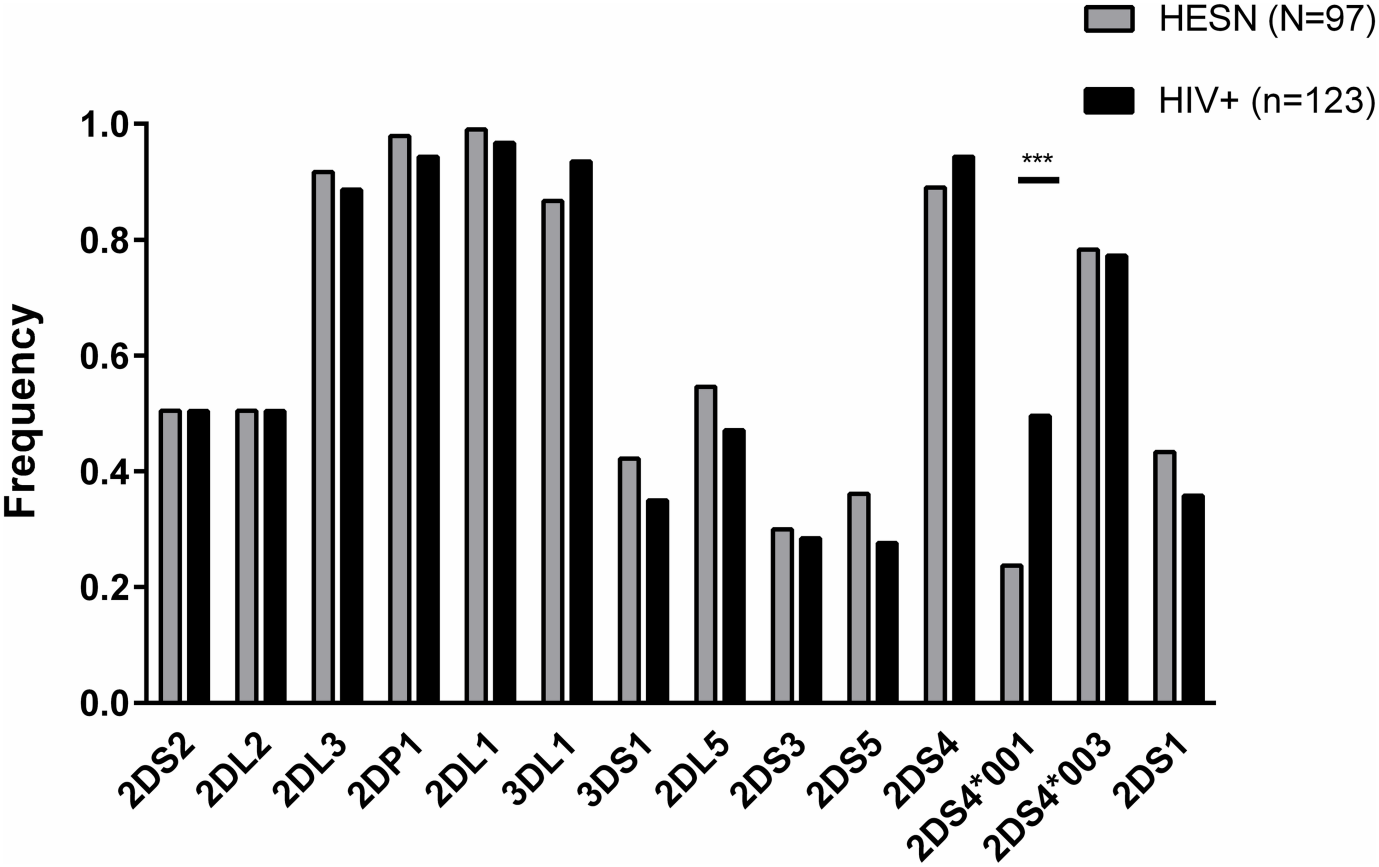
Killer Immunoglobulin-like Receptor (KIR) gene and KIR allele group frequencies in 97 HIV exposed seronegative (HESN) and 123 recently infected HIV positive (HIV+) subjects. Shown on the y-axis are the percentage of HESN and HIV+ individuals carrying each KIR gene. Percentage refers to the number of subjects positive for each variable divided by the total number of subjects tested for that variable. The framework genes *KIR2DL4*, *KIR3DL2*, *KIR3DL3*, and the pseudogenes *KIR3DP1* were present in all study subjects and are not shown in this this figure. Each gene shown on the x-axis is named without the “KIR” designation, i.e. 2DS1 = KIR2DS2, etc. ** = p’<0.01. This p-value refers to p-value corrected for multiple comparisons. This p’-value is shown over the bar linking the 2 groups being compared.

The KIR region is polygenic and thus varies in gene content from one individual to another [9, 11]. To determine whether the frequency of certain KIR genes within this region differed between HESN and HIV+ subjects we screened for their presence in a subset of 97 HESN and 123 HIV+ individuals by *KIR* gene region typing. The frequency of *KIR2DL2* and *KIR3DL3* was considered separately as was the frequency of *KIR3DL1* and *KIR3DS1*, which are allele groups at the *KIR2DL2/L3* and *KIR3DL1/S1* loci, respectively. We also considered the presence of a *KIR2DS4* gene, and whether alleles at this locus belonged to the KIR2DS4*001-like and/or KIR2DS4*003-like groups. As expected, the framework genes *KIR2DL4*, *KIR3DL2*, and *KIR3DL3* and the pseudogene *KIR3DP1* were present in all subjects tested. Fig 1 and S1 Table show the frequency of each of these genes and allele groups in HESN and HIV+ subjects. The only significant between-group differences noted was the frequency of KIR2DS4*001-like alleles, which was lower in HESN than in HIV+ subjects (0.33 (0.18, 0.59), p = 0.0002, corrected p (p’) = 0.003, Fisher’s exact test). The frequency of several other telomeric KIR genes differed between HESN and HIV+ subjects, though these differences did not achieve statistical significance. This prompted us increase the size of the study population to provide increased power to observe significant between-group differences in telomeric *KIR* gene frequency.

**Fig 2:**
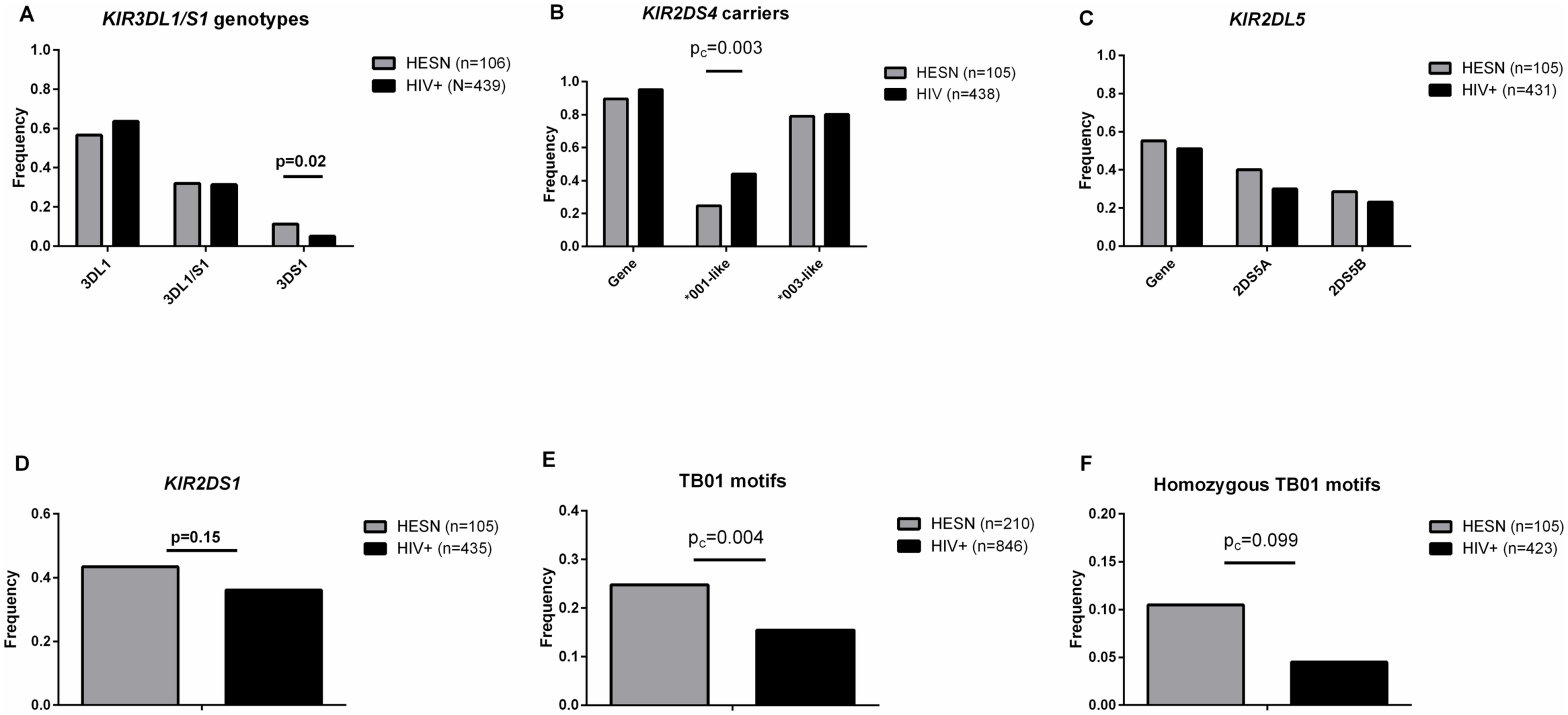
Killer Immunoglobulin-like Receptor (KIR) generic genotype and allele group frequencies in HIV exposed seronegative (HESN) and recently infected HIV positive (HIV+) subjects. Shown on the y-axis is the frequency of (A) HESN (n = 106) and HIV+ (n = 439) subjects positive for the three *KIR3DL1/S1* generic genotypes, (B) HESN (n = 105) and HIV+ (n = 438) subjects positive for a *KIR2DS4* gene and carrying at least 1 copy of a KIR2DS4*001-like or KIR2DS4*003-like allele (C) KIR2DS4*001-like or KIR2DS4*003-like allele groups among the 210 and 876 KIR haplotypes from HESN (n = 105) and HIV+ (n = 438) subjects, (D) HESN (n = 105) and HIV+ (n = 431) subjects positive for a *KIR2DL5*, *KIR2DL5A* and *KIR2DL5B* gene, (E) HESN (n = 105) and HIV+ (n = 435) positive for a *KIR2DS1* gene, (F) *TB01* motifs among the 210 and 846 KIR haplotypes from HESN (n = 105) and HIV+ (n = 423) subjects and (G) HESN (n = 105) and HIV+ (n = 423) subjects positive for a homozygous *TB01* motif. P’-values over the lines linking groups being compared are corrected for multiple comparisons.

We previously showed that the distribution of the *KIR3DL1/S1* generic genotypes were in Hardy-Weinberg equilibrium in HIV+ but not in HESN subjects [23]. The skewed distribution of *KIR3DL1/S1* generic genotypes in HESN was due to an over representation of *KIR3DS1* hmz among HESN. This observation was confirmed in a larger group of 106 HESN and 439 HIV+ subjects, however the significance of this finding did not survive correction for multiple comparisons (Fig 2A and S2 Table).

Fewer HESN than HIV+ individuals carried a *KIR2DS4* gene, though the statistical significance of this difference did not survive correction for multiple comparisons (p = 0.03, p’ = 0.36, Fisher’s) (Fig 2B, S2 Table). Fewer HESN than HIV+ subjects carried at least 1 copy of a full length KIR2DS4*001-like allele, which encodes cell surface expressed receptors that have the potential to exert an effect on NK cell function. There were no significant between-group differences in the frequency of carriage of unexpressed KIR2DS4*003-like alleles (Fig 2B, S2 Table). *KIR2DS4* and *KIR2DS1* are genes in the telomeric *KIR* region that are in strong negative LD suggesting and that may be alleles at the same locus. Based on whether a *KIR2DS1* gene was also present it was possible to deduce whether 1 or 2 copies of full length KIR2DS4*001-like or truncated KIR2DS4*003-like alleles were present in subjects that typed for only one of these allele groups. As shown in Fig 2C and S2 Table the frequency of KIR2DS4*001-like alleles, was significantly lower in HESN than in HIV+ persons (p<0.001, p’-value p = 0.01, Fisher’s) while that of KIR2DS4*003-like alleles did not differ between groups. The frequency of *KIR2DS1* carriers did not differ significantly between groups (1.38 [0.89, 2.13] p = 0.15, Fisher’s) (Fig 2D and S2 Table). The proportion of HESN and HIV+ individuals positive for a *KIR2DL5* gene was not significantly different (p = 0.82, Fisher’s, Fig 2E, and S2 Table). A *KIR2DL5* gene can be present in either centromeric or telomeric group B *KIR* haplotypes. Although the frequency of telomeric *KIR2DL5A* was higher in HESN than HIV+ subjects, this difference did not achieve statistical significance. (Fig 2E and S2 Table).

In summary, we found that absence of an expressed KIR2DS4*001-like allele was associated with a reduced risk of HIV infection and confirmed a trend towards an association between *KIR3DS1* homozygosity and a reduced risk of HIV infection.

### The telomeric *KIR3DS1*, *KIR2DL5A*, *KIR2DS3/5* and *KIR2DS1* gene grouping is more frequent in HESN than HIV+ subjects

**Fig 3:**
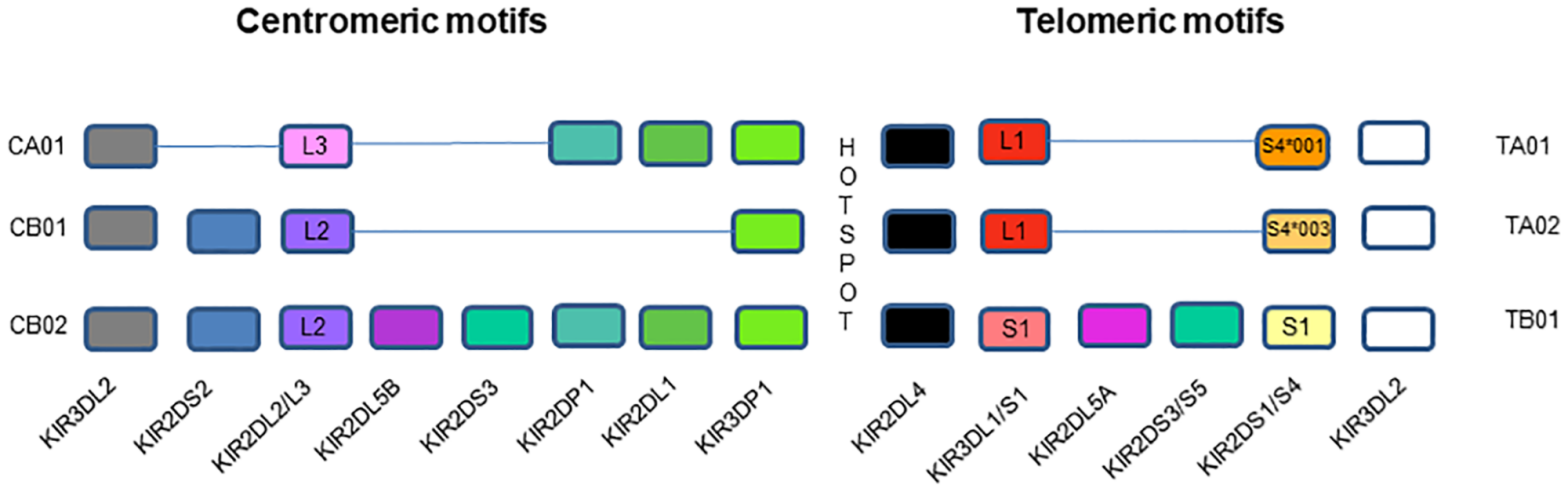
Killer Immunoglobulin-like (KIR) haplotypes. Organization and composition of centromeric and telomeric KIR region genes.

The most common KIR haplotypes are derived from combinations of three centromeric and three telomeric motifs linked to each other by a recombination hotspot located between *KIR3DP1* and *KIR2DL4* [17, 40, 41]. Fig 3 shows that *KIR3DS1* is positioned within the telomeric group B haplotype *TB01* motif, in LD with *KIR2DL5A*, *KIR2DS3/S5* and *KIR2DS1* [17, 40, 41].

HESN (n = 105) and HIV+ (n = 423) subjects were typed for *KIR3DL1/S1* genotypes, and for the presence of *KIR2DL5A*, *KIR2DS1* and *KIR2DS4* genes. All subjects positive for *KIR3DS1*, *KIR2DL5A* and/or *KIR2DS1* were also typed for the presence of *KIR2DS3* and *KIR2DS5*, one of which should also be present in *TB01* motifs. While most study subjects carried canonical *TA01/02* and/or *TB01* motifs, 6 (5.7%) HESN and 58 (13.7%) HIV+ subjects carried non-canonical telomeric KIR motifs. S3 Table provides information on the number of individuals who carried non-canonical motifs and identifies how they diverged from canonical telomeric motifs. All the non-canonical telomeric motifs were classified as non *TB01*.

Of the 210 and 846 telomeric *KIR* region motifs in 105 HESN and 423 HIV+ subjects 52 (24.8%) and 126 (14.9%), respectively were canonical *TB01* motifs, a proportional between-group difference that was statistically significant (p = 0.0003, p’ = 0.004, Fisher’s) (Fig 2F and S2 Table). HESN were more likely to carry a homozygous *TB01* motif than HIV+ subjects (11 of 105 [10.48%] HESN versus 15 of 423 [3.5%] HIV+ persons were *TB01* homozygotes [3.18 (1.42, 7.15), p = 0.009, Fisher’s). However, the significance of this between-group difference did not survive correction for multiple comparisons (Fig 2G and S2 Table).

### The contribution of *TB01* encoded gene products to NK cell responses to HLA-null cell stimulation

**Fig 4:**
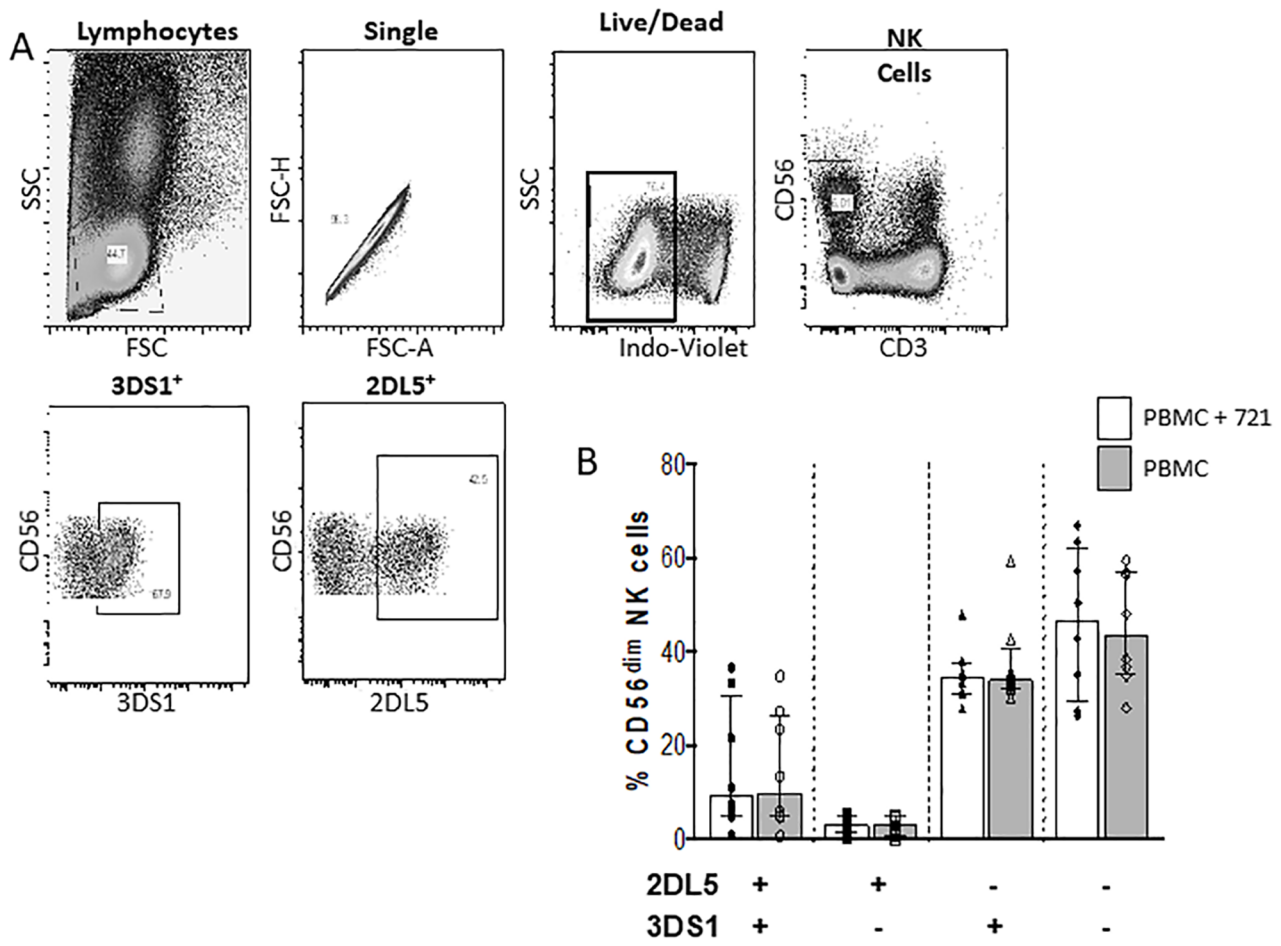
The frequency of NK cells expressing all possible combinations of KIR3DS1 and KIR2DL5 before and after stimulation with 721.221 (221) HLA-null cells. (A) Live singlet lymphocytes were gated on. From this population CD3^-^CD56^dim^ NK cells were examined for the frequency of cells expressing KIR3DS1 and/or KIR2DL5 or neither. (B) The frequency of NK cells on the y-axis expressing all possible combinations of KIR3DS1 (3DS1) and KIR2DL5 (2DL5) on NK cells before and after stimulation with 221 cells (B). Each point represents results from a single individual. Bar heights and error bars represent the median and inter-quartile range of each group.

Although the *KIR3DS1*, *KIR2DL5A*, *KIR2DS1* and, when present, *KIR2DS5* genes are in LD they are stochastically expressed on NK cell populations. This prompted us to question the contribution of NK cells expressing various combinations of these receptors to stimulation with 221 cells. Of 8 *KIR3DS1* hmz, all were positive for *KIR2DL5A* and *KIR2DS1* and 6 were positive for *KIR2DS5*. NK cells from these individuals were investigated for functional responses to 221 cell stimulation. Fig 4A shows the gating strategy used to identify KIR3DS1^+^ and KIR2DL5^+^ NK cell populations that were stained with panel 1 antibodies. We used Boolean analysis to examine the frequency of the 4 possible NK populations defined by KIR3DS1^+/-^ KIR2DL5^+/-^ receptors before and following stimulation with 221 cells (Fig 4B). No significant difference in the frequency of expression of the 4 NK cell populations was noted indicating that HLA-null stimulation did not alter the expression of these KIR on the NK cell surface.

**Fig 5:**
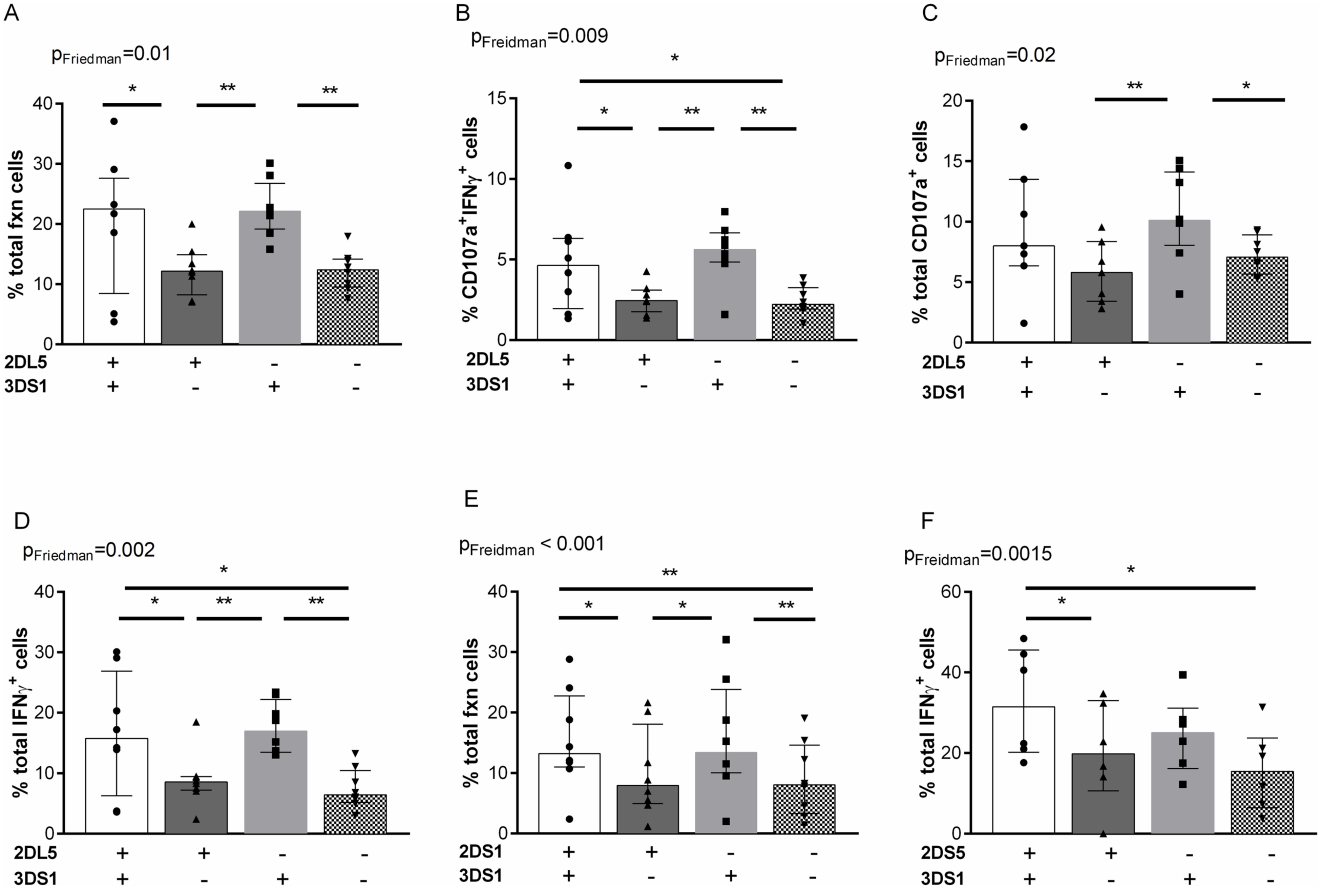
The frequency functional NK cells expressing all possible combinations of KIR3DS1 and KIR2DL5, KIR3DS1 and KIR2DS1 and KIR3DS1 and KIR2DS5 responding to stimulation with 721.221 (221) HLA null cells. Shown in the y-axis are the frequencies of NK cells expressing (A-D) all possible combinations of KIR3DS1 (3DS1) and KIR2DL5 (2DL5) (E) 3DS1 and KIR2DS1 (2DS1) and (F) 3DS1 and KIR2DS5 (2DS5) that responded to stimulation with 221 cells. (A, E) Frequency of all functional NK cells (% total fxn cells), (B) NK cells secreting both IFN-γ and CD107a, (% CD017a+IFN-γ+ cells), (C) all NK cells expressing CD107a (% total CD107a+ cells) (D, F), and all NK cells secreting IFN-γ (% total IFN-γ cells). Each point represents results from an individual subject. Bar heights and error bars represent the median and inter-quartile range of each group. Lines linking 2 bars show comparisons between the groups linked by the lines. * = p<0.05, ** = p<0.01.

We also used Boolean analysis to ascertain the functional responses to 221 cell stimulation characterized by degranulation, as measured by CD107a, and IFN-γ secretion of the 4 possible NK populations defined by KIR3DS1^+/-^ KIR2DL5^+/-^ receptor expression (S1 Fig). The frequency of the double positive (3DS1^+^2DL5^+^) and KIR3DS1^+^ (3DS1^+^2DL5^-^) NK cells responding to 221 stimulation was significantly higher than that of the single KIR2DL5^+^ (3DS1^-^2DL5^+^) and double-negative (3DS1^-^2DL5^-^) NK cell populations (Fig 5). This was the case for all the functional subsets examined (Fig 5A, 5B and 5D), with the exception of the total CD107a^+^ functional profile, where between-group comparison did not achieve statistical significance (Fig 5C). There was no significant difference in the frequency of responding cells characterized by any of the functional subsets for the double positive and single 3DS1^+^ NK cell populations. A higher frequency of 3DS1^+^2DL5^+^ than 2DL5^+^ NK cells responded to 221 stimulation for all functional subsets except total CD107a expression. The frequency of single 2DL5^+^ and double-negative 3DS1^-^2DL5^-^ NK cells responding to 221 stimulation was not significantly different for any of the functional subsets examined.

**Fig 6:**
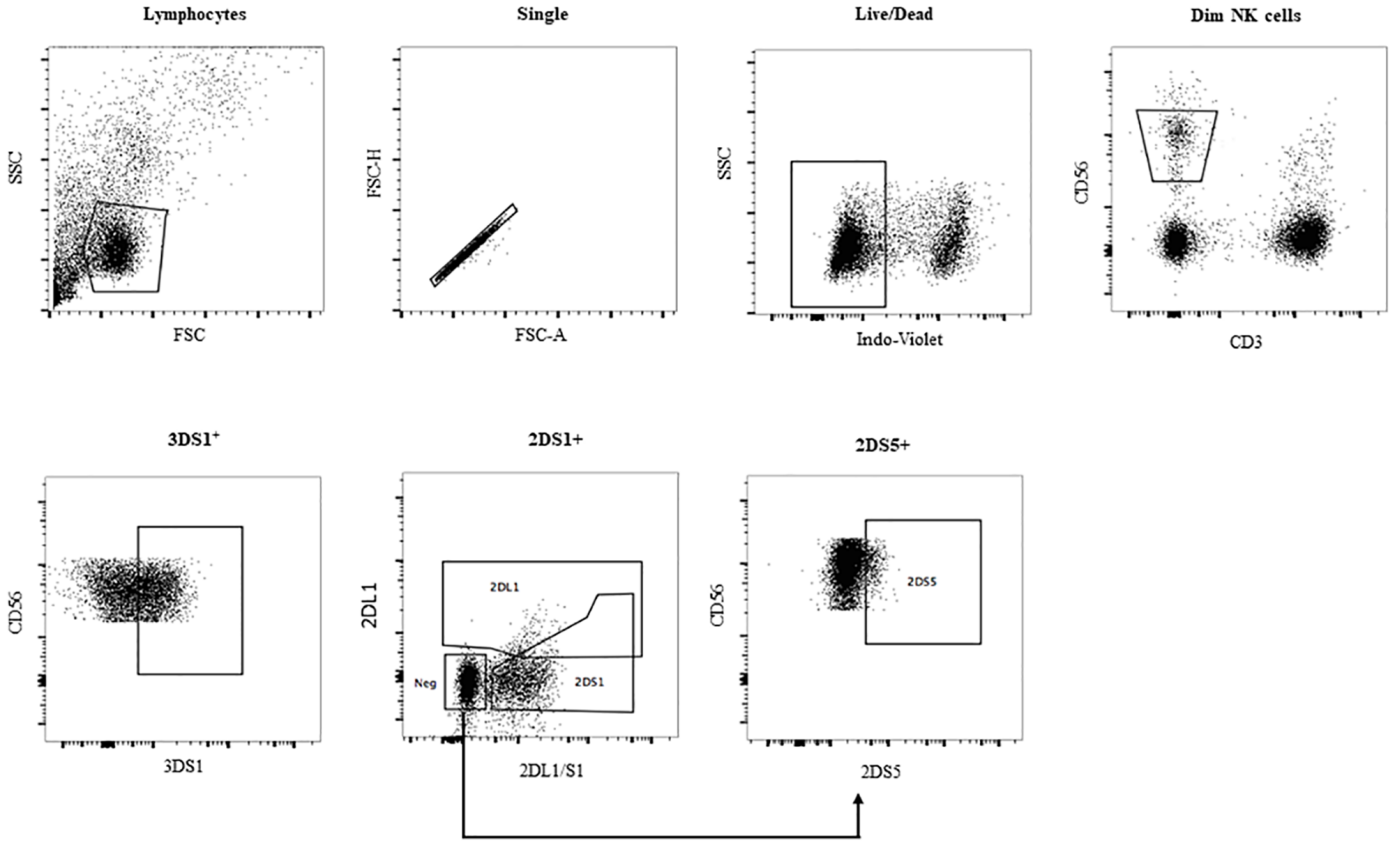
The gating strategy used to determine the frequency of NK cells expressing KIR3DS1, KIR2DS1 and KIR2DS5. Live singlet lymphocytes were gated on. From this population, CD3^-^CD56^dim^ NK cells were identified from which the frequency of NK cells expressing KIR3DS1 (3DS1) was assessed. CD3^-^CD56^dim^ NK cells were also gated on to determine the frequency of cells expressing KIR2DS1 (2DS1). This was accomplished by using the monoclonal antibody (mAb) REA284 specific for KIR2DL1 (2DL1) only, to bind this KIR making it unavailable for recognition by a second mAb (11PB6) conjugated to a different fluorochrome that was specific for both 2DL1 and 2DS1. This strategy permitted the separation of 2DL1^+^2DS1^-^ NK cells from 2DL1^-^2DS1^+^ cells and 2DL1^-^2DS1^-^ NK cells [42]. To detect KIR2DS5^+^ NK cells, the CD3^-^CD56^dim^ 2DL1^-^2DS1^-^ NK cells from the previous gate that bound to mAb HP-MA4 specific for 2DL1/2DS1/KIR2DS3 (2DS3)/KIR2DS5 (2DS5) were gated on. Since 2DS3 is not cell surface expressed, this mAb detected only 2DS5^+^ NK cells among those negative for 2DL1 and 2DS1.

Fig 6 shows the gating strategy used to identify KIR3DS1^+^, KIR2DS1^+^ and KIR2DS5^+^ NK cells following staining with panel 2 antibodies. Boolean analysis was used to examine the frequency of the 4 possible NK populations defined by KIR3DS1^+/-^KIR2DS1^+/-^ and by KIR3DS1^+/-^KIR2DS5^+/-^ expression and for ascertaining the frequency of functional NK cells responding to 221 stimulation by expressing CD107a and secreting IFN-γ within each of these phenotypic NK cell populations (S1 Fig). A higher frequency of double and single KIR3DS1^+^ than KIR3DS1^-^ NK cells responded to stimulation by exhibiting the sum of all functions tested whether KIR3DS1 was expressed with KIR2DS1 or not (Fig 5E). This was also the case for total IFN-γ secretion. Single KIR2DS1^+^ (3DS1^-^2DS1^+^) NK cells responded no better to 221 stimulation than did double negative 3DS1^-^2DS1^-^ NK cells. A higher frequency of single and double KIR3DS1^+^ NK cells than KIR3DS1^-^ NK cells responded to stimulation by secreting IFN-γ whether KIR3DS1 was expressed with KIR2DS5 (2DS5) or not (Fig 5F). Single KIR2DS5^+^ (3DS1^-^2DS5^+^) NK cells responded no better to 221 stimulation than double negative 3DS1^-^2DS5^-^ NK cells.

Together, these results support the conclusion that a higher frequency of KIR3DS1^+^ than KIR3DS1^-^ NK cells responded to 221 cells and indicate that KIR3DS1^+^ NK cells contributed more to NK functional responses to HLA-null cell stimulation than did KIR2DL5A^+^, KIR2DS1^+^ or KIR2DS5^+^.

## Discussion

HESN, compared to HIV+ subjects, were more likely to carry 1 or 2 copies of a telomeric *TB01* motif and were less likely to carry a full length KIR2DS4*001-like allele. As most *KIR3DS1* hmz were also *KIR2DL5A*, *KIR2DS1* and *KIR2DS5* positive we investigated the functional potential of NK cells expressing various combinations of the KIR receptors encoded by these genes to stimulation with the HLA null cell line 221. Functional studies revealed that NK cells expressing KIR3DS1 alone or with any one of the KIR2DL5A, KIR2DS1 or KIR2DS5 receptors responded better to 221 stimulation than NK cells expressing one of these KIRs alone without KIR3DS1 or none of these receptors. These results suggest that of the KIR receptors encoded by genes present within *TB01* motifs, KIR3DS1 contributes most to NK cell responsiveness to 221 HLA null cells.

NK cells from subjects with no *KIR2DS4* gene, or who carry only KIR2DS4*003-like alleles do not express this receptor on their cell surface. On the other hand, NK cells from carriers of KIR2DS4*001-like alleles do express a KIR2DS4 receptor on a subset of their NK cells. Merino et al. reported that expressed KIR2DS4 was associated with poor outcome in the context of HIV infection such as higher viral load, HIV transmission in HIV discordant couples and low CD4 counts [24, 25]. This observation was confirmed by Olvera et al. in an HIV+ population from Lima, Peru and may have depended on the co-carriage of HLA-Cw4, a presumed ligand for this receptor [26]. Our results suggest that expressed KIR2DS4 is also associated with reduced resistance to HIV infection. The mechanisms underlying these outcomes are not understood. KIR2DS4 is an aKIR that is the product of gene conversion with the *KIR3DL2* gene that has led to a reduced ability to recognize HLA-C ligands characteristic of KIR2D receptors and an increased ability to recognize HLA-A*11:02 and HLA-A*03 ligands, the presumed ligands for KIR3DL2 [43]. Further investigations are needed to understand whether, and if so how, KIR2DS4 expression is associated with negative outcomes in the context of HIV infection and HIV exposure. The possibility that the impact of expression of KIR2DS4 on poor HIV outcomes is due to other genes in LD with KIR2DS4 has not been excluded.

HESN cohorts have been studied to identify mechanisms underlying resistance to HIV. In a cohort of Vietnamese HESN injection drug users (IDU), NK cells were found to be more active than those from HIV uninfected persons who eventually seroconverted [44]. A study comparing 25 HESN with 19 HIV+ IDU and 26 HIV uninfected persons found that HESN had KIR and KIR/HLA expression profiles consistent with a lower threshold of NK cell activation and higher ratios of *KIR3DS1*:*KIR3DL1* transcripts [45]. A higher prevalence of *KIR3DS1* or lower frequency of *KIR3DL1* in HESN than HIV susceptible subjects has been reported in several studies, and this, often in the absence of an association with HLA-Bw4 [23, 45–48]. Overall, these studies support the interpretation that HIV resistance may be due to NK cells that are more easily activated, which is consistent with carriage of a group B *KIR* haplotype in which larger numbers of genes encoding aKIR are present.

A study of HIV discordant and concordant couples found a role for alloreactive NK cells in protection from sexual HIV transmission [49]. The implication that alloreactive NK cells may play a role in HIV resistance could not be investigated here because the HIV+ index partner to which HESN and HIV+ subjects in this study were exposed is unknown. However, these studies highlight mechanisms other than carriage of aKIR that may be responsible for HIV susceptibility/resistance. For example, carriage of *KIR3DL1* high expression genotypes with HLA-B*57 has been associated with a reduced risk of HIV infection in HESN and NK cells from individuals carrying this KIR/HLA combination are particularly responsive to stimulation by HLA-null cells and by autologous HIV infected cells. NK cells from carriers of this KIR/HLA combination have a superior ability to inhibit HIV replication compared to those from carriers of the receptor or ligand alone or neither [22, 50, 51].

*KIR* genes in the telomeric and centromeric *KIR* regions are present in LD with each other. This confounds the identity of the KIR gene product responsible for effects on HIV outcomes. One would expect that if *KIR* genotypes influence HIV exposure outcome they would do so through their effects on NK cell function. One way to test for NK cell functional potential is to stimulate with HLA null cells. When this was done with NK cells from *KIR3DS1* hmz who co-carried *KIR2DL5A*, *KIR2DS1 and KIR2DS5*, but no *KIR2DS4*, genes the NK cells that responded with the highest frequency were KIR3DS1^+^. Co-expression of KIR2DL5, KIR2DS1 or KIR2DS5 did not modulate the frequency of responding KIR3DS1^+^ NK cells nor did KIR2DL5^+^, KIR2DS1^+^ and KIR2DS5^+^ NK cells respond to this stimulus any better that their KIR2DL5^-^, KIR2DS1^-^ or KIR2DS5^-^ counterparts. In these study subjects, expression of KIR2DS4 would not have played a role in NK cell responsiveness since none carried a *KIR2DS4* gene. Although some subjects were positive for a *KIR2DL5B* gene the alleles they carried at this locus encoded unexpressed gene products, eliminating any role for KIR2DL5B^+^ NK cells in responses to 221 cells [32, 39]. The contribution of KIR2DS3 to NK cell functionality is likely limited by its low expression on the cell surface [52]. Together these findings support the notion that, of the *KIR* genes in *TB01* motifs, *KIR3DS1* encodes a receptor that confers NK cells with functionality, at least to HLA-null 221 cells.

HLA-F has recently been identified as a ligand for KIR3DS1 [53]. HLA-F is present on the surface of 221 cells, which may explain why these cells stimulate KIR3DS1^+^ NK cells. Attempts to find ligands for KIR3DS1 among HLA-Bw4 or among the HLA-Bw4 alleles having an isoleucine at position 80 of the HLA heavy chain (HLA-Bw4*80I) have largely failed [54–56]. An exception to this are results reported by O’Connor et al. showing that KIR3DS1 can interact with the HLA-Bw4*80I antigen HLA-B*57 when certain peptides, including some HIV derived peptides, are present. [57]. However, if such interactions were contributing to NK cell education they should tune down KIR3DS1^+^ NK cell responsiveness to 221 stimulation. Thus, it is more likely that the interactions between KIR3DS1 and HLA-F is responsible for NK cell stimulation by 221 cells. Since HLA-F is usually intracellular in resting cells it would not be expected to contribute to NK cell education and tune down the responsiveness of KIR3DS1^+^ NK cells. However, it is cell surface expressed on activated cells, including HIV-infected CD4^+^ T cells. The interaction of KIR3DS1 on NK cells with HLA-F in HIV infected cells may explain why KIR3DS1^+^ NK cells are superior to KIR3DL1^+^ NK cells in suppressing HIV replication [53, 58]. This interaction may provide a mechanistic explanation for the association of *KIR3DS1* with protection from HIV infection [53]. More needs to be done to explore the role of KIR3DS1/HLA-F interactions in the recognition of HIV infected cells.

The ligand for KIR2DL5 has not been identified, though this receptor is likely an iKIR based this receptor having intra-cellular ITIM motifs [30]. Other iKIR usually participate in NK cell education resulting from their interaction with HLA antigen ligands during NK cell development. Education is required for the development of functional potential and the transmission of inhibitory signals to NK cells required for tolerance to self. These inhibitory signals are interrupted when the ligand for iKIR are absent, which occurs in the setting of several viral infections or cellular transformation. KIR2DS1 interacts with HLA-C2 group antigens, though with a lower affinity than its inhibitory counterpart KIR2DL1 [59]. It can participate in NK cell education, but tunes down NK cell activation potential if present with an HLA-C2 ligand, presumably to avoid reactivity to self [60]. KIR2DS5 is cell surface expressed and appears to transmit activating signals when cross linked with antibodies binding this receptor [61]. However, the identity of it ligand is unknown.

The background level of functionality in the single positive KIR2DL5^+^, KIR2DS1^+^ and KIR2DS5^+^ and double negative NK cell populations may either be due to co-expression of other iKIR such as KIR2DL1/L2/L3 participating in NK cell education in subjects co-carrying HLA-C ligands for these receptors or to inhibitory NK cell receptors such as NKG2A, which interacts with ubiquitously expressed HLA-E complexed with epitopes from the leader sequence of several HLA antigens [20, 62–64]. These receptor ligand interactions would be expected to be more or less evenly distributed among the 4 KIR3DS1^+/-^/KIR2DL5^+/-^ subsets as well as among the 4 KIR3DS1^+/-^KIR2DS1^+/-^ and KIR3DS1^+/-^KIR2DS5^+/-^ subsets.

One of the limitations of this study is that autologous HIV infected cells have not been used to stimulate NK cell populations defined by various combinations of their *TB01* motif encoded gene products. Although we know that HIV infected cells express HLA-F, the ligand for KIR3DS1 it is possible, though as yet unknown, whether they express ligands for KIR2DL5, KIR2DS1 and/or KIR2DS5 that could account for NK cell functionality that plays a role in HIV control. This possibility requires further exploration.

In summary, despite this limitation, the higher frequency of *TB01* motifs among HESN than HIV+ subjects studied here would be consistent with the interpretation that their resistance to HIV infection is related to their NK cells having a lower activation threshold due to the presence of aKIRs whose genes map to this region and by expressing the KIR3DS1 receptor able to mediate activating signals upon interacting with HLA-F on HIV infected CD4 T cells and HLA-null 221 cells. This does not explain resistance to HIV in all HESN. Thus, further investigation is needed to uncover other possible mechanisms underlying reduced susceptibility to HIV infection.

## Supporting information

S1 Fig The gating strategy for obtaining the frequency of NK cells expressing all possible combinations of KIR3DS1 (3DS1) and/or KIR2DL5 (2DL5) secreting IFN-γ and/or expressing CD107a or neither. (TIF)

S1 Table Comparison of the frequency of KIR genes and allele groups in HESN and HIV infected subjects. (DOCX)

S2 Table Comparison of the frequency of KIR genes in HESN and HIV infected subjects. (DOCX)

S3 Table Description of deviations from canonical telomeric KIR motifs. (DOCX)
