# Supplementary figures and images for: HIV exposed seronegative (HESN) compared to HIV infected individuals have higher frequencies of telomeric Killer Immunoglobulin-like Receptor (KIR) B motifs; Contribution of KIR B motif encoded genes to NK cell responsiveness
Source: PLoS One. 2017 Sep 22;12(9):e0185160. doi: 10.1371/journal.pone.0185160 (PMC5609756; doi:10.1371/journal.pone.0185160)

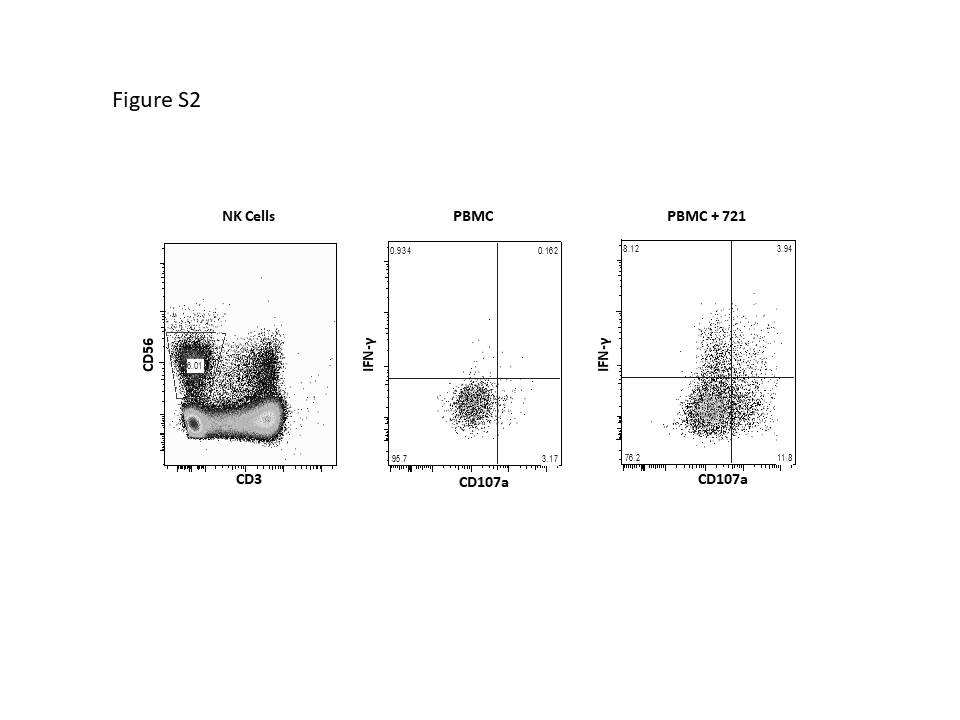

Supplement: S1 Fig — (TIF) [file pone.0185160.s001.tif]
