# Supplementary material for: HIV exposed seronegative (HESN) compared to HIV infected individuals have higher frequencies of telomeric Killer Immunoglobulin-like Receptor (KIR) B motifs; Contribution of KIR B motif encoded genes to NK cell responsiveness
Source: PLoS One. 2017 Sep 22;12(9):e0185160. doi: 10.1371/journal.pone.0185160 (PMC5609756; doi:10.1371/journal.pone.0185160)
Supplement: S1 Table — (DOCX) [file pone.0185160.s002.docx]

**S1_Table.** Comparison of the frequency of KIR genes and allele groups in HESN and HIV infected subjects.

| **Gene**  **/allele group** | **HESN**  **(n=97)** | **HIV+**  **(n=123)** | **p-value** | **OR (CI)** |
| --- | --- | --- | --- | --- |
| KIR2DS2 | 49 (50.5)^a^ | 62 (50.4)^a^ | 1 | 1 (0.59, 1.7) |
| KIR2DL2 | 49 (50.5) | 62 (50.4) | 1 | 1 (0.59, 1.7) |
| KIR2DL3 | 89 (91.7) | 109 (88.6) | 0.5 | 1.4 (0.57, 3.56) |
| KIR2DP1 | 95 (97.9) | 116 (94.3) | 0.085 | 0.23 (0.05, 1.15) |
| KIR2DL1 | 96 (99.0) | 119 (96.7) | 0.39 | 3.23 (0.35, 29.4) |
| KIR3DL1 | 86 (88.7) | 115 (93.5) | 0.23 | 0.74 (0.49, 1.12) |
| KIR3DS1 | 41 (42.2) | 43 (35.0) | 0.33 | 1.18 (0.88, 1.6) |
| KIR2DL5 | 43 (54.6) | 118 (47.1) | 0.28 | 1.35 (0.79, 2.3) |
| KIR2DS3 | 29 (30.0) | 35 (28.4) | 0.76 | 1.12 (0.62, 2) |
| KIR2DS5 | 35 (36.1) | 34 (27.6) | 0.19 | 1.5 (0.83, 1.62) |
| KIR2DS4 | 86 (89.0) | 116 (94.3) | 0.14 | 0.47 (0.17, 1.25) |
| KIR2DS4*001-like^b^ | 23 (23.7) | 61 (49.6) | <0.001 | 0.32 (0.18, 0.57) |
| KIR2DS4*003-like^b^ | 76 (78.3) | 95 (77.2) | 0.87 | 1.1 (0.56, 2.0) |
| KIR2DS1 | 42 (43.3) | 44 (35.8) | 0.27 | 1.37 (0.79, 2.36). |

^a^ Results presented as number (percent)

^b^ Carriers of at least 1 full length KIR2DS4*001-like or truncated *003-like allele among 97 HESN and 123 HIV+ subjects..

HESN =HIV Exposed Seronegative; OR = Odds Ratio; CI = 95% confidence intervals.
