# Supplementary material for: HIV exposed seronegative (HESN) compared to HIV infected individuals have higher frequencies of telomeric Killer Immunoglobulin-like Receptor (KIR) B motifs; Contribution of KIR B motif encoded genes to NK cell responsiveness
Source: PLoS One. 2017 Sep 22;12(9):e0185160. doi: 10.1371/journal.pone.0185160 (PMC5609756; doi:10.1371/journal.pone.0185160)
Supplement: S2 Table — (DOCX) [file pone.0185160.s003.docx]

**S2 Table.** Comparison of the frequency of KIR genes in HESN and HIV infected subjects.

| **Genotype**  **/locus/motif** | **HESN** | **HIV+** | **p-value**  **(p’)^a^** | **OR (CI)** |
| --- | --- | --- | --- | --- |
| KIR3DL1 hmz^b^ | 60 (56.6)^b^ | 279 (63.6)^c^ | 0.22 | 0.75 (0.48, 1.52) |
| KIR3DL1/S1 het^b^ | 34 (32.1) | 138 (31.4) | 1.0 | 1.0 (0.63, 1.57) |
| KIR3DS1 hmz^b^ | 12 (11.3) | 22 (5.0) | 0.024  (0.24) | 2.4 (1.56, 5.06) |
| KIR2DS4^d^ | 94 (89.5) | 417 (95.2) | 0.036  (0.32) | 0.2.3 (1.08, 5.0) |
| KIR2DS4*001-like carriers^d^ | 26 (24.8) | 193 (44.4) | 0.0002  (0.003) | 0.49 (0.32, 0.75) |
| KIR2DS4*003-like carriers^d^ | 83 (59.0) | 351 (55.0) | 0.79 | 0.94 (0.55, 1.52) |
| KIR2DS4*001-like alleles^e^ | 29 (13.8) | 214 (24.5) | 0.0009  (0.011) | 0.44 (0.28, 0.68) |
| KIR2DS4*003-like alleles^e^ | 124 (59.0) | 482 (55.0) | 0.31 | 1.18 (0.87, 1.6) |
| KIR2DL5^f^ | 58 (55.2) | 216 (50.1) | 0.38 | 1.23 (0.8, 1.89) |
| KIR2DL5A^f^ | 42 (40) | 147 (34.9) | 0.26 | 1.23 (0.83, 2.0) |
| KIR2DL5B^f^ | 30 (28.6) | 108 (25.1) | 0.45 | 1.2 (0.74, 1.93) |
| KIR2DS1^g^ | 46 (43.8) | 157 (36.0) | 0.15 | 1.38 (0.9, 2.13) |
| TB01 motif^h^ | 52 (24.8) | 126 (14.9) | 0.0003  (0.004) | 2.0 (1.39, 2.9) |
| TB01 hmz motif^i^ | 11 (10.48) | 15 (3.5) | 0.009  (0.099) | 3.18 (1.42, 7.15) |

^a^ Holm corrected p-value.

^b^ For analyses on *KIR3DL1/S1* generic genotyping, 106 HESN and 439 HIV+ subjects were included.

^c^ Results presented as number (percent) positive for variable.

^d^ For analyses on *KIR2DS4* genes and carriers of at least 1 *KIR2DS4*001*-like or at least 1 *KIR2DS4*003*-like allele, 105 HESN and 438 HIV+ subjects were included.

^e^ For analyses on the number of full length *KIR2DS4*001*-like or truncated *KIR2DS4*003*-like alleles 210 and 876 genes present in 105 HESN and 438 HIV+ subjects were included.

^f^ For analyses on KIR2DL5 typing 105 HESN and 431 HIV+ subjects were included.

^g^ For analyses on KIR2DS1 typing 105 HESN and 435 HIV+ subjects were included.

^h^ For analyses on the number of *TB01* motifs in 210 and 846 haplotypes from 105 HESN and 423 HIV+ subjects were included.

^i^ For analyses on *TB01* hmz motifs, 105 HESN and 423 HIV+ subjects were included.

HESN=HIV Exposed Seronegative Subjects; OR = Odds Ratio; CI = 95% confidence intervals.
