# Supplementary material for: HIV exposed seronegative (HESN) compared to HIV infected individuals have higher frequencies of telomeric Killer Immunoglobulin-like Receptor (KIR) B motifs; Contribution of KIR B motif encoded genes to NK cell responsiveness
Source: PLoS One. 2017 Sep 22;12(9):e0185160. doi: 10.1371/journal.pone.0185160 (PMC5609756; doi:10.1371/journal.pone.0185160)
Supplement: S3 Table — (DOCX) [file pone.0185160.s004.docx]

**S3 Table. Description of deviations from canonical telomeric KIR motifs.**

|  | HESN  n | HIV+  n |
| --- | --- | --- |
| Number (percent) of carriers of non-canonical motifs | 6  (5.7) | 58 (13.7) |
| Absence of a *KIR2DS1* gene in what would otherwise be a *TA01/TB01* heterozygous motifs | 2 | 18 |
| Presence of a *KIR2DS1* gene in what would otherwise be a *TA01* homozygous motifs | 3 | 17 |
| Absence of a *KIR2L5A* gene what would otherwise be a *TA01/TB01* heterozygous motif | 1 | 5 |
| Presence of a *KIR2DL5A* gene in what would other wise be a *TA01* homozygous motif | 0 | 5 |
| Absence of *KIR2DS3* and *KIR2DS5* genes in what would otherwise be a *TA01/TB01* heterozygous motifs | 0 | 6 |
| Absence of *KIR2DS1* and *KIR2DL5A* in what would otherwise be a *TA01/TB01* heterozygous motifs | 0 | 3 |
| Absence of *KIR2DS1*, and *KIR2DS3* or *KIR2DS5* genes in what would otherwise be a *TA01/TB01* heterozygous motifs | 0 | 1 |
| Absence of *KIR2DL5A* and *KIR2DS3* or *KIR2DS5* genes in what would otherwise be a *TA01/TB01* heterozygous motifs | 0 | 1 |
| Absence of *KIR2DS1*, *KIR2DL5A*, *KIR2DS3* or *KIR2DS5* genes in what would otherwise be a *TA01/TB01* heterozygous motifs | 0 | 1 |
